# Investigation of Interface Interactions Between Monolayer MoS_2_ and Metals: Implications on Strain and Surface Roughness

**DOI:** 10.1021/acs.langmuir.3c02740

**Published:** 2024-01-03

**Authors:** Jz-Yuan Juo, Klaus Kern, Soon Jung Jung

**Affiliations:** † Max-Planck-Institut Für Festkörperforschung, Heisenbergstraße 1, Stuttgart DE-70569, Germany; ‡ École Poly Technique Fédérale de Lausanne, Institut de Physique, Lausanne CH-1015, Switzerland

## Abstract

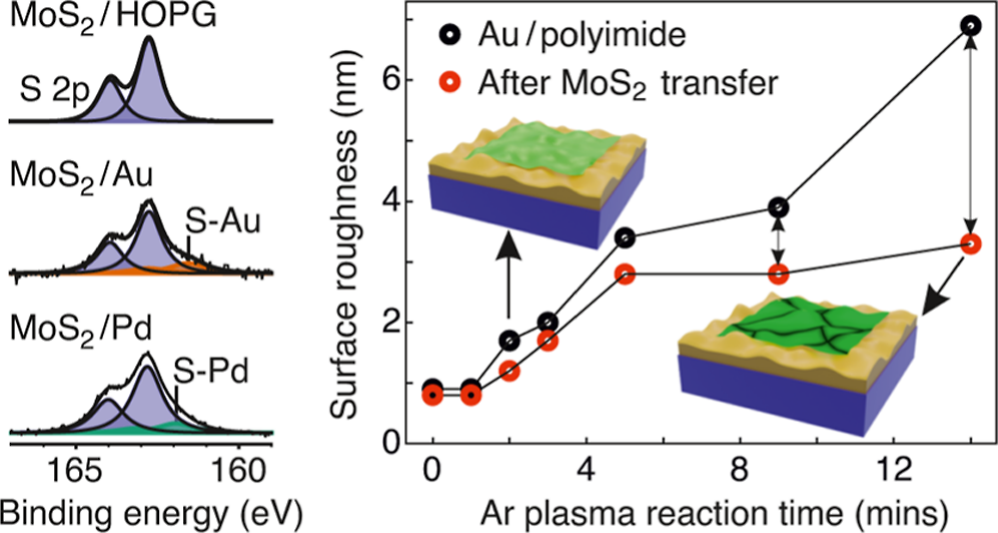

Achieving a low contact resistance has been an important issue in the design of two-dimensional (2D) semiconductor–metal interfaces. The metal contact resistance is dominated by interfacial interactions. Here, we systematically investigate 2D semiconductor–metal interfaces formed by transferring monolayer MoS_2_ onto prefabricated metal surfaces, such as Au and Pd, using X-ray photoelectron spectroscopy (XPS), atomic force microscopy, and Raman spectroscopy. In contrast to the MoS_2_/HOPG interface, the interfaces of MoS_2_/Au and MoS_2_/Pd feature the formation of weak covalent bonds. The XPS spectra reveal distinct peak positions for S–Au and S–Pd, indicating a higher doping concentration at the S–Au interface. This difference is a key factor in understanding the electronic interactions at the metal–MoS_2_ interfaces. Additionally, we observe that the metal surface roughness is a critical determinant of the adhesion behavior of transferred monolayer MoS_2_, resulting in different strains and doping concentrations. The strain on transferred MoS_2_ increases with an increase in substrate roughness. However, the strain is released when the roughness of metal surface surpasses a certain threshold. The dependence of the contact material and the influence of the substrate roughness on the contact interface provide critical information for improving 2D semiconductor–metal contacts and device performance.

## Introduction

One of the major challenges of semiconductor device research is the contact between the semiconductor and metal electrodes for the efficient injection of charge carriers into the conduction channel. Therefore, understanding and designing the semiconductor–metal interface has been an important issue,^1^ underpinning the performance of transistors,^2^ batteries,^3^ and catalysis.^4^ Moreover, as the dimensions of electronic devices scale down to sub-20 nm, the metal contact resistance starts to dominate the total device resistance.^5^ Particularly, for two-dimensional (2D) semiconductors, the contacts of 2D devices usually possess a large Schottky barrier and rarely follow the Schottky–Mott model, because of interfacial effects.^6^ As high parasitic contact resistance has been identified as a critical limiting factor in the performance of transition-metal dichalcogenides (TMdCs)-based devices, the understanding of interface properties between 2D materials and metals is critical.^7^

Extensive research into the interaction between 2D TMdCs and metal electrodes has been conducted. For metals that are prone to oxidation, such as Ir, Cr, Sc, Ti, and Y, the interaction with the S atoms is strong,^8−10^ and alters the atomic structure of the MoS_2_, resulting in extensive disorder. For inert metals, such as Au, it has been shown that the Au interacts with the MoS_2_ through van der Waals forces^11−15^ or covalent-like quasi-bonding.^16^ Although the intrinsic MoS_2_ structure is not affected through contact with Au, the orbital hybridization between MoS_2_ and Au was still observed, leading to modifications of the bandgap, conductivity, and surface reactivity of MoS_2_.^12−14,17,18^

However, the reported values of contact resistance and charge carrier injection efficiency vary from sample to sample and depend on the measurement method.^7^ One of the reasons for this is the extreme sensitivity of 2D materials to surface adsorbates or environments, which can seriously limit the realization of Ohmic contacts in 2D devices.^19−21^ The complicated interfacial states induced by defects, adsorbates, orbital hybridization, or chemical disorders can also be easily formed at contact interfaces.^22^ Furthermore, surface morphology plays a critical role in determining contact resistance between the 2D semiconductor and metal. Surface roughness, atomic arrangement, and the presence of defects can all influence the quality of the contact and the efficiency of charge carrier injection. The atomically thin nature of 2D materials results in their crystal structure being easily damaged during standard device fabrication processing, such as e-beam lithography^23^ and physical vapor deposition of metals,^24^ resulting in a significant Schottky barrier or interfacial states. Strain is another critical extrinsic stimulus. Strain is inevitable in 2D materials, regardless of whether the film is suspended^25^ or supported.^26^ Furthermore, strain is known to alter the physical and chemical properties, such as the band gap,^26^ charge carrier effective masses,^27^ dielectric properties,^28^ chemical reactivity,^29^ and so on. The local strain induced at the 2D-metal interface might be more complex and varied compared to that on traditional dielectric substrates due to the increased interface interactions after transfer.^30−32^ For example, at the interface between MoS_2_ and Au, the combination of charge and strain induces the 2H-to-1T phase transition of MoS_2_,^33^ which largely changes the properties of monolayer MoS_2_ from semiconducting to metallic.

In our study, we aim to investigate the dependence of the contact material in conjunction with the interface roughness. The contact interfaces between monolayer MoS_2_ and inert metals, such as Au and Pd were studied using X-ray photoelectron spectroscopy (XPS), Raman spectroscopy, and atomic force microscopy (AFM). These metals were chosen, as they are the most widely used contact materials. The experiment was carefully designed to isolate and examine only the intended effects while excluding any other potential influences. The 2D semiconductor–metal interfaces are formed by transferring monolayer MoS_2_ onto prefabricated metal surfaces to avoid disorders induced by the fabrication of metal contacts.^24^ This method also reduces the presence of multilayer regions, commonly seen in exfoliation techniques, which could affect data interpretation.^34,35^ Furthermore, the interfaces are easily accessed without the limitation of metal film thickness since MoS_2_ is on top of the metal substrate.^9,34^ We use MoS_2_ transferred on HOPG as a reference so that the contact material effects can be distinguished from the other effects, such as defects and grain boundaries. Our XPS analysis reveals that when monolayer MoS_2_ is brought in contact with Au or Pd, additional interface states emerge due to the metal–S interaction. We further study the roughness dependence of interface states by transferring monolayer MoS_2_ onto prefabricated metal surfaces with different roughness. Through a combined analysis of Raman spectra and AFM images, we observe an increase in strain on MoS_2_ with higher metal surface roughness. However, once the roughness exceeds a certain threshold, the strain and doping concentration on MoS_2_ decrease due to delamination. Furthermore, XPS spectra indicate that the positions of the interface states are less affected by strain but are more sensitive to the degree of doping concentration.

## Results and Discussion

**Figure 1:**
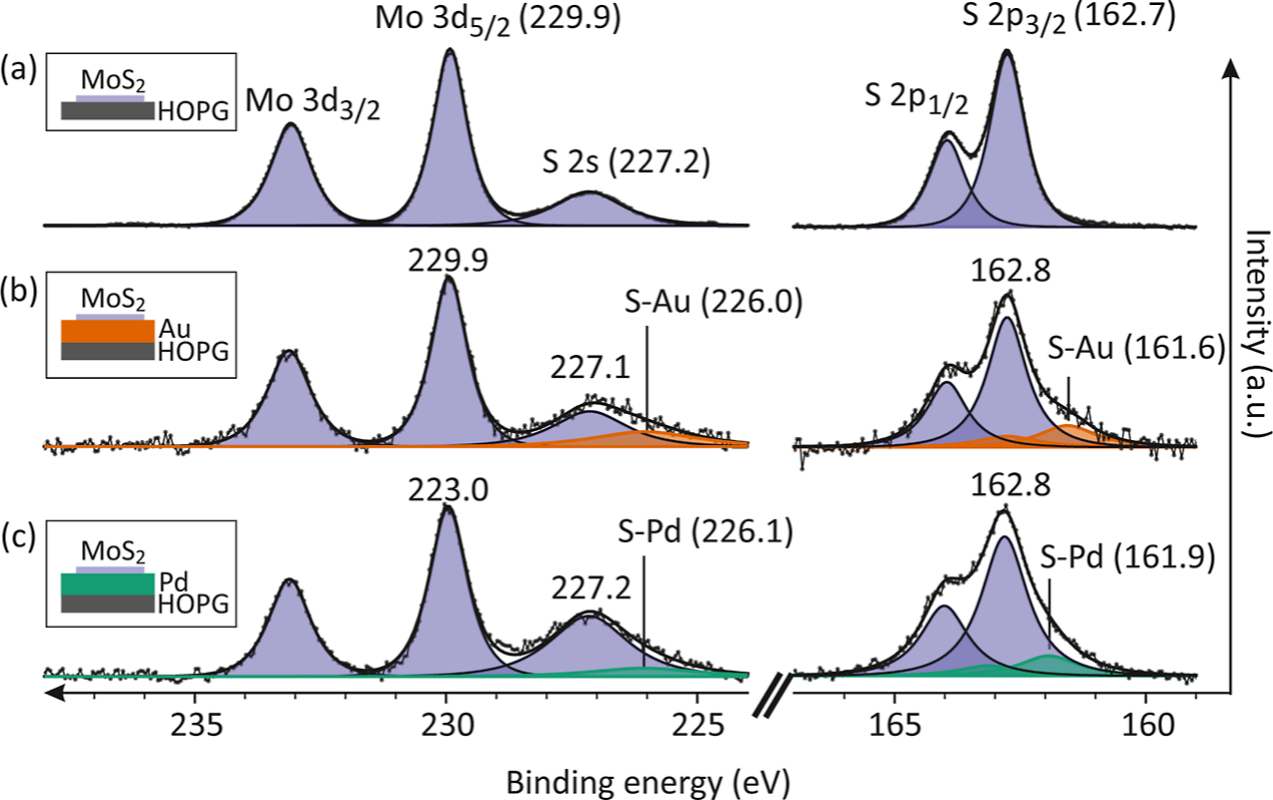
MoS_2_/Au and MoS_2_/Pd interfacial interaction. Normalized XPS spectra of the Mo 3d, S 2s, and S 2p core levels of monolayer MoS_2_ on three different substrates: (a) HOPG, (b) Au-deposited HOPG, and (c) Pd-deposited HOPG. Peaks from MoS_2_ are marked as purple. S 2s (2p) peaks for the S–Au and S–Pd interactions are marked as orange and green, respectively.

To exclude the effect of substrate roughness, we use highly oriented pyrolytic graphite (HOPG) as the substrate, upon which the metal film with a thickness of 50 nm is deposited. Since we used a bottom contact structure, with the metallic substrate beneath the MoS_2_ layer, X-ray photoelectron spectroscopy (XPS) could be used to characterize the interfacial interaction. As shown in Figure 1, three different MoS_2_–metal interfaces were studied by transferring monolayer MoS_2_ onto: HOPG (MoS_2_/HOPG), Au-deposited HOPG (MoS_2_/Au/HOPG), and Pd-deposited HOPG (MoS_2_/Pd/HOPG).

Figure 1a shows the core-level spectra of MoS_2_/HOPG with visible peaks of Mo 3d_3/2_ (233.1 eV), Mo 3d_5/2_ (229.9 eV), S 2s (227.2 eV), S 2p_1/2_ (163.9 eV), and S 2p_3/2_ (162.7 eV), consistent with previous findings.^36^ Using the relative sensitivity factor of 5.77 for Mo 3d_5/2_ and 1.25 for S 2s from CasaXPS library,^37^ the Mo:S ratio is found to be ∼1:2. Together with the absence of Mo-oxide doublet peaks (236 eV),^38^ it suggests that our sample has a low defect concentration and is not degraded during the transfer process.

Figure 1b shows the spectra for MoS_2_/Au/HOPG, both S 2p and S 2s spectra are broad compared to the peaks of the MoS_2_/HOPG spectra.^12,13,39,40^ The broadening is attributed to the emergence of new S–Au peaks in S 2s and S 2p in MoS_2_/Au/HOPG, which show a uniform shift toward lower binding energies compared to those of the MoS_2_/HOPG film. The new S 2p and S 2s appear at 161.6 and 226.0 eV, ∼1.2 and ∼1.1 eV lower than that of the S–Mo peak in S 2p and S 2s spectra, respectively. There is almost no obvious difference in the binding energies of Mo 3d peaks between MoS_2_/HOPG and MoS_2_/Au/HOPG. Similar peaks were observed in MoS_2_/Pd/HOPG (Figure 1c). S–Pd peaks were observed at 161.9 and 226.1 eV, which have less separation to the S–Mo peak in S 2p and S 2s spectra than that of the S–Au peaks.

The presence of additional S–Au peaks is attributed to the metal–S interaction at the interface. The interaction between metal and the sulfur atoms of MoS_2_ establishes a weak covalent bond, leading to an accumulation of electrons around the sulfur atoms.^1,6,14,17,20,41−43^ This electron accumulation, as evidenced by the shift in the XPS peak positions, contributes to the reduction in binding energy.^44^ The observed differences in binding energy shifts between the S–Pd and S–Au peaks can be primarily attributed to the varying degrees of electron accumulation at these interfaces. Table 1 presents our Raman measurements of doping concentrations that corroborate our XPS findings. Notably, the S–Au interface exhibits a higher n-type doping concentration, evidenced by a larger shift in the XPS peak compared to the S–Pd interface. On the other hand, the broadening of Mo peaks is also found because of the simultaneous environment change of Mo atoms. The fwhm of Mo 3d_3/2_ and Mo 3d_5/2_ peaks of MoS_2_/Au/HOPG and MoS_2_/Pd/HOPG is increased from ∼0.9 and ∼0.8 eV to ∼1.1 and ∼1.0 eV, respectively, which aligns well with the shift of the S 2s and S 2p peaks. However, assigning new components of the Mo 3d peak was not possible due to the limited XPS resolution.

**Table 1:** Summary of the Parameters Extracted From Raman Spectra Measured on Monolayer MoS_2_/Au and MoS_2_/Pd Interfaces Under Different Substrate Conditions^a^

	Ar plasma		Raman spectra		
sample	time (mins)	E’ (cm^–1^)	A_1_’ (cm^–1^)	strain (%)	N-type doping (10^12^ cm^–2^)
MoS_2_/Au/PI #1	0	385.1 ± 0.5	404.2 ± 0.2	0.16	9.4
MoS_2_/Au/PI #2	1	384.6 ± 0.4	403.8 ± 0.2	0.25	10.4
MoS_2_/Au/PI #3	2	384.2 ± 0.4	403.8 ± 0.2	0.33	9.8
MoS_2_/Au/PI #4	3	384.0 ± 0.5	404.7 ± 0.1	0.37	5.6
MoS_2_/Au/PI #5	5	385.2 ± 0.2	405.3 ± 0.2	0.13	4.7
MoS_2_/Au/PI #6	9	385.2 ± 0.2	404.8 ± 0.2	0.13	5.1
MoS_2_/Au/PI #7	14	385.9 ± 0.1	405.4 ± 0.1	0	5.0
MoS_2_/Au/HOPG		382.5 ± 0.9	402.8 ± 0.8	0.65	12.0
MoS_2_/Pd/PI #1	0	384.2 ± 0.3	404.7 ± 0.1	0.32	5.6
MoS_2_/Pd/PI #2	1	383.8 ± 0.2	404.8 ± 0.1	0.41	5.0
MoS_2_/Pd/PI #3	1.5	385.2 ± 0.3	405.0 ± 0.1	0.14	5.8
MoS_2_/Pd/PI #4	2	385.1 ± 0.1	405.0 ± 0.2	0.15	5.7
MoS_2_/Pd/PI #5	9	385.3 ± 0.3	405.2 ± 0.2	0.11	5.1
MoS_2_/Pd/HOPG		382.3 ± 0.4	404.6 ± 0.2	0.70	3.2

a Polyimide (PI).

It was reported that defects^45−47^ and phase transition^48−52^ can also trigger peaks emerging at lower binding energy shoulders in XPS spectra. To verify this, we perform Raman spectroscopy to examine the defect density and whether the main features of the 1T′ octahedral structure exist, as shown in Figure S1. The absence of LA(M) mode gives an upper bound on the defect density of <10^13^ cm^–2^ in our sample condition (interdefect distance < 3.2 nm).^53^ Moreover, except for E′ and A_1_^′^ peaks, the other three characteristic peaks of the 1T phase at ∼157 (J_1_), ∼224 (J_2_), and ∼320 cm^–1^ (J_3_) are not observed. Therefore, we exclude the defects and the phase transition as the origin of the new peaks.

**Figure 2:**
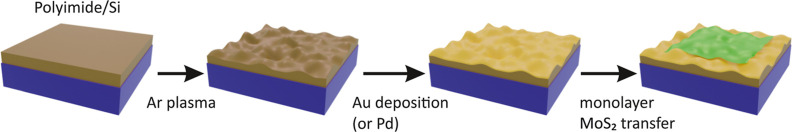
Schematic of our method to investigate monolayer MoS_2_/metal interactions with increasing interface roughness. The monolayer MoS_2_ is transferred onto the Au-deposited or Pd-deposited polyimides treated with an increasing Ar plasma time.

To elucidate the surface roughness effect on the MoS_2_–metal interface, we used a polyimide/Si substrate. A polyimide substrate was chosen because the surface roughness can be controlled via exposure to Ar plasma. As shown in Figure 2, the polyimide film supported by the silicon substrate is exposed to Ar plasma, which roughens the surface, with a controlled reaction time ranging from 0 to 14 min. Then, Au or Pd films with a thickness of ∼50 nm were evaporated on the as-treated polyimide film with the increased surface roughness. Finally, the monolayer MoS_2_ is transferred onto the metal-coated polyimide films using a wet etching method.^54^ The following experiments are performed on both MoS_2_/Au/polyimide and MoS_2_/Pd/polyimide. Since both experiments point to the same conclusion, we present the results for Au in the paper and the results for Pd in the Supporting Information.

**Figure 3:**
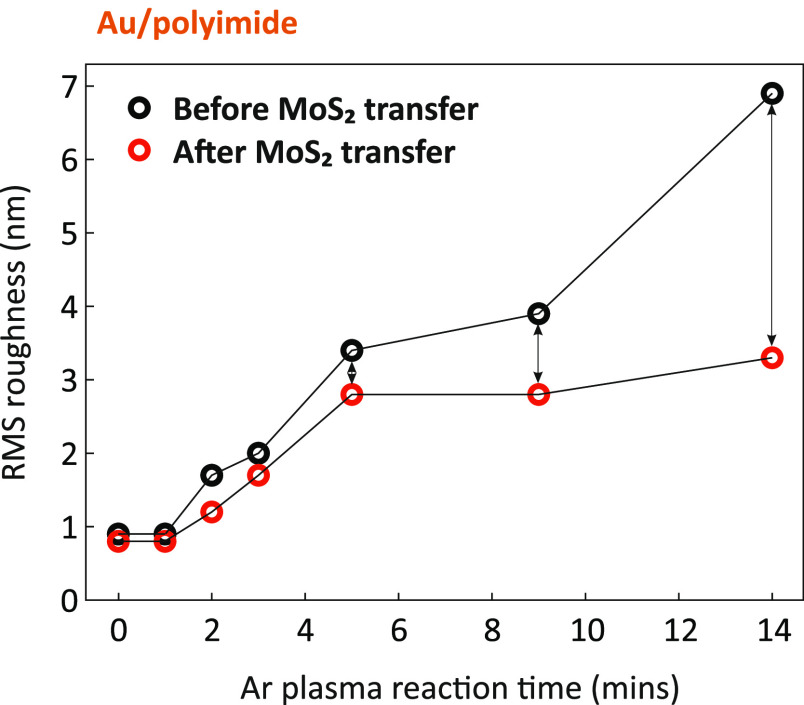
Root-mean-square (RMS) surface roughness of Au-deposited polyimide with increasing Ar plasma reaction time before and after transferring monolayer MoS_2_.

Figure 3 shows the root-mean-square (RMS) surface roughness of Au/polyimide and MoS_2_ Au/polyimide with increasing Ar plasma reaction time, which is calculated from AFM topography images (AFM figures are in Figure S2). RMS roughness of both surfaces has a positive correlation with Ar plasma reaction time. However, the difference in roughness between the two films increases as the Ar plasma reaction time increases. As the Ar plasma time increases, the roughness of Au/polyimide continues to increase, while that of MoS_2_/Au/polyimide does not increase significantly and is almost constant from 5 min onward.

**Figure 4:**
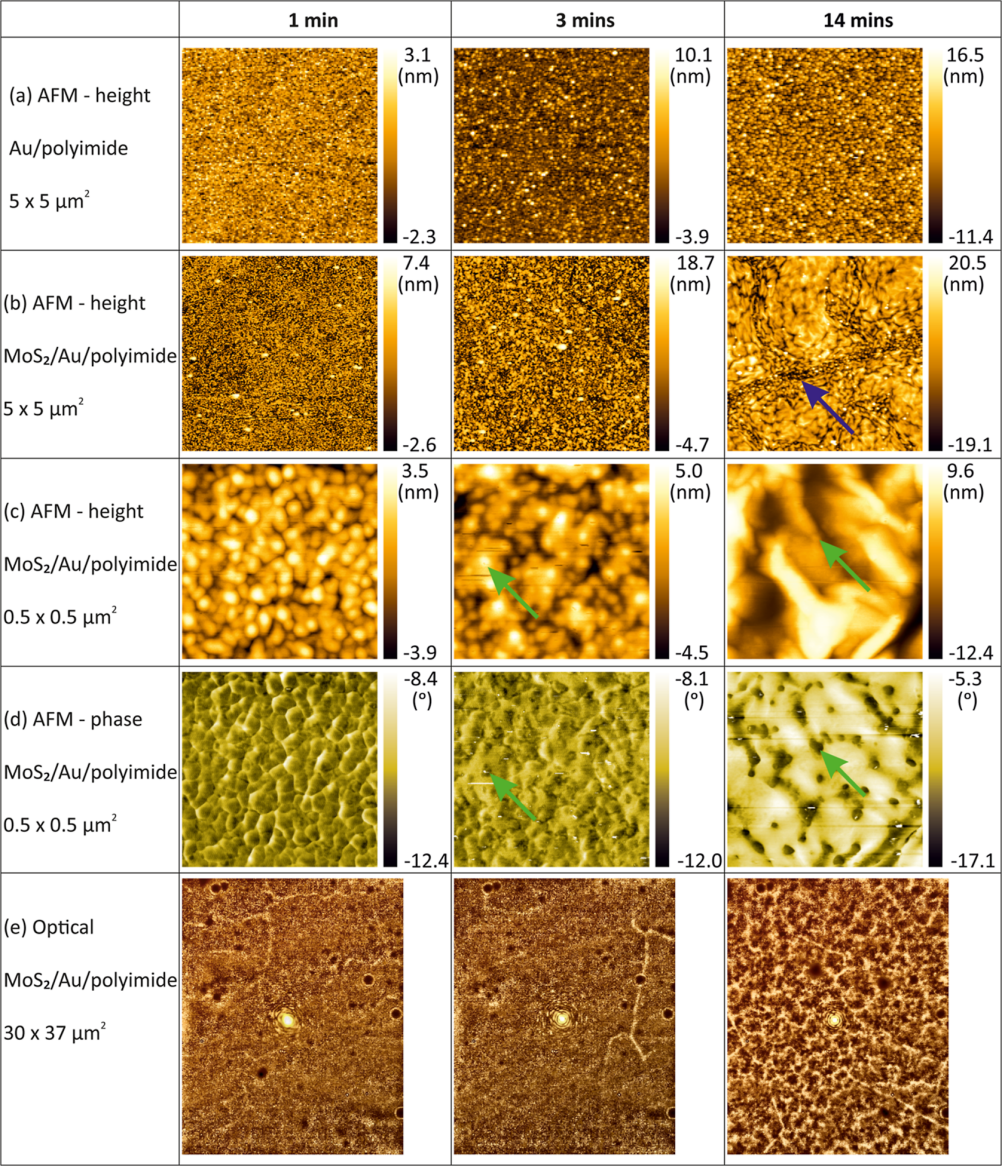
Representative AFM topography images of Au/polyimide (a) before and (b) after transferring the monolayer MoS_2_ with increasing Ar plasma reaction time from 1, 3, to 14 min. Higher resolution AFM (c) topography and (d) simultaneously measured phase images of MoS_2_/Au/polyimide. (e) Optical images. Focused green laser spots are centered in each image.

To map the topography of the Au/polyimide before and after transfer of MoS_2_, as shown in Figure 4a, tapping mode AFM was used. When 50 nm Au film is evaporated on the polyimide surface, it forms a homogeneous granular structure. After transferring MoS_2_ on the Au surface (Figure 4b,c), the surface structure does not follow the granular structure of the Au film but shows an inhomogeneous surface structure. The difference in morphology before and after MoS_2_ transfer is more pronounced as the polyimide substrate becomes rougher, consistent with the results in Figure 3. Figure 4c,d shows enlarged AFM topography and phase images. Phase imaging in AFM captures the phase shift signal of the cantilever oscillation in tapping mode, which is sensitive to variation in material composition, adhesion, friction, viscoelasticity, as well as other factors.^55^ As shown in Figure 4d, the morphology change of the MoS_2_ surface is more clearly visible in the phase images. As plasma treatment time increased more than 3 min, black dot-like features emerged. The black dots in the phase image are located at protruded areas in the topography image, where the MoS_2_ is strongly contacted to the Au film (marked with green arrows, see overlay in Figure S3). The density of these areas reduces as the plasma duration increases, reflecting a reduction in the MoS_2_ area that is intimately coupled to the metal surface, i.e., more of the MoS_2_ is suspended. This suspension of MoS_2_ on metal surfaces was previously reported when MoS_2_ is exfoliated by metal surfaces.^35^ In contrast to the MoS_2_/Au/polyimide morphology, the phase images of Au/polyimide show only granular features and do not change even as plasma duration is increased (Figure S4). We also observed cracks formed after MoS_2_ transfer onto the rough surface after 14 min of plasma treatment on polyimide, marked with the purple arrow in Figure 4b (an enlarged image is shown in Figure S5). Inside the cracks, we can see the exposed metal film with a granular structure. The density of these cracks increases with surface roughness, as shown in the optical microscopy image in Figure 4e.

**Figure 5:**
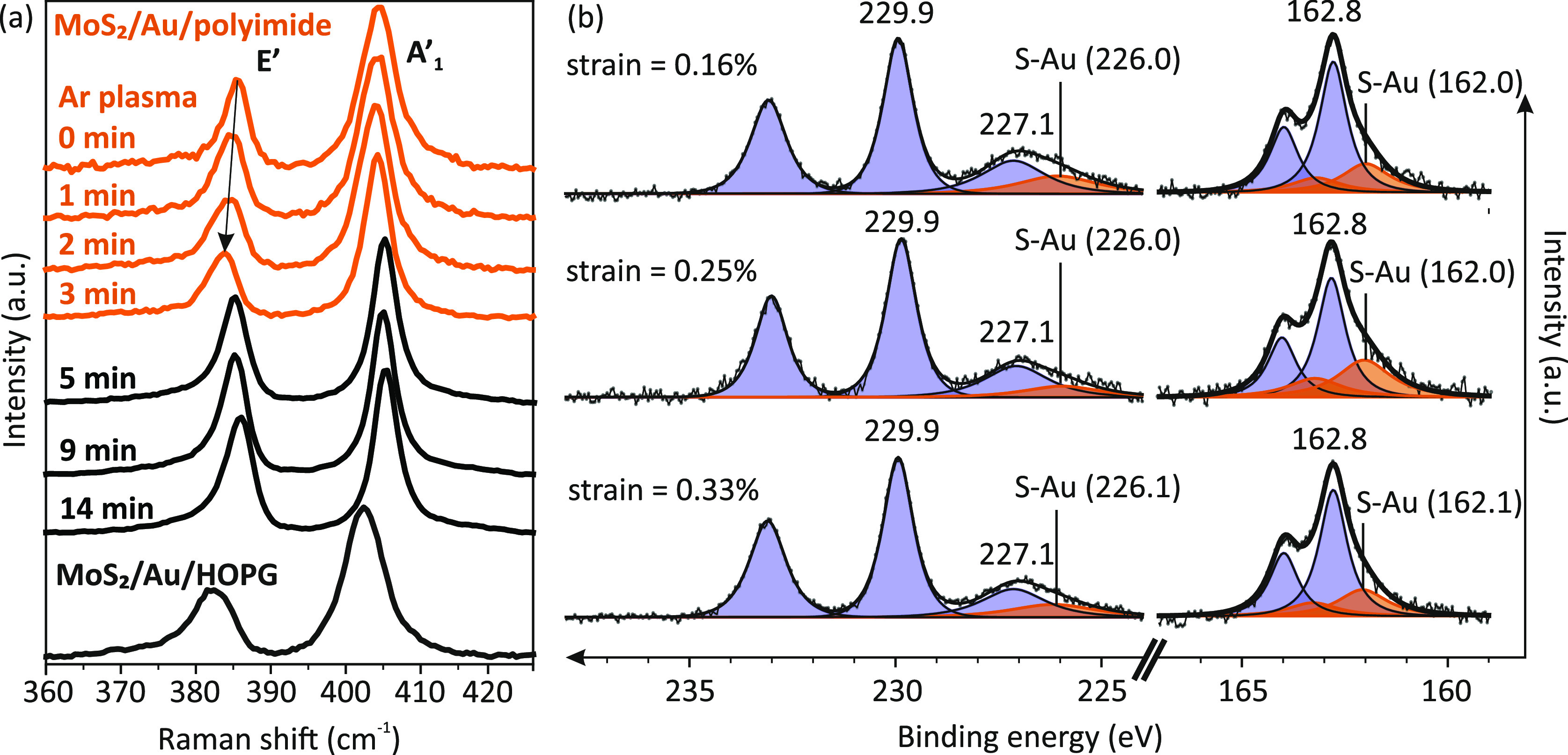
Strain dependence of MoS_2_/Au interfacial interaction. (a) Normalized representative Raman spectra of monolayer MoS_2_/Au/polyimides treated with increasing Ar plasma reaction time. (b) Normalized XPS spectra of the Mo 3d, S 2s, and S 2p core levels of monolayer MoS_2_/Au/polyimide with increasing strain, which are extracted from their Raman spectra, as shown in Table 1. Peaks from intrinsic MoS_2_ are marked as purple. S 2s and S 2p peaks for S–Au are marked as orange.

Having demonstrated the dependence of the morphology change of transferred MoS_2_ on the substrate roughness, we used Raman spectroscopy to characterize its effect on the interface interaction. Figure 5a shows the normalized Raman spectra of monolayer MoS_2_/Au/polyimide with an increasing Ar plasma reaction time. For the case without treatment of Ar plasma (0 min), the in-plane E′ and the out-of-plane A_1_′ vibrational modes were observed at 385.1 and 404.2 cm^–1^, respectively. The intensity is normalized by the A_1_′ peak. For each condition, 10 spots on each sample were measured and averaged. The value of 385.9 cm^–1^ is used as our zero-strain reference (ref (35)). The strain is measured by using shifts in the E′ peak with its linear relationship of −5.2 cm^–1^/% to strain.^56^ For estimation of doping concentration, the strain-induced peak shift of A_1_′ mode is first corrected by the linear relationship (1.7 cm^–1^/%) and then the doping concentration is calculated by its linear relationship (−2.2 cm^–1^/10^13^ cm^–2^) to A_1_′ peak shift,^57^ where the peak at 406.5 cm^–1^ in the MoS_2_/HOPG is used as our zero-doping reference.

Strain and doping values extracted from the Raman spectra are summarized in Table 1. For MoS_2_/Au/polyimide, the strain increases from 0.16 to 0.37% when Ar plasma reaction time increases from 0 to 3 min. However, when the Ar plasma reaction time is increased by more than 3 min, the strain falls to below 0.13%. Interestingly, the doping concentration also reduces from ∼1 × 10^13^ to ∼6 × 10^12^ cm^–2^ when the Ar plasma reaction time reaches 3 min. This is consistent with AFM results in Figures 3 and 4, which indicates that the MoS_2_ film is partially suspended after 3 min plasma treatment (Figure 4c). This suspended area, which is separated from the Au film with a larger contact distance, may be the origin of the reduction of doping concentration (Table 1). When MoS_2_ is transferred to a rougher surface, the monolayer is broken and partially delaminated from the underlying metal surface, resulting in strain relaxation.

We utilized XPS to characterize the strain dependence of the metal–S interactions (Figure 5b). The XPS measurements are conducted on MoS_2_/Au/polyimide samples with varying surface roughness. To mitigate any edge effects, we ensured that the surface roughness remained below the delamination threshold. Strain values are determined from individual Raman spectra, as summarized in Table 1. As strain increases from 0.16 to 0.33%, the separation between the S–Au and S–Mo peaks in the S 2p (S 2s) spectra decreases from 0.8 (1.1) to 0.7 (1.0) eV. Given that the peak shift is approximately 0.1 eV, which is close to the precision of our fitting, it is challenging to definitively attribute this shift to strain effects. However, it is worth noting that strain can alter bond distances and angles, leading to charge redistribution.^58−60^

The experiment is repeated for MoS_2_/Pd/polyimide, pointing to the same conclusion, as shown in Figures S6–S9. Strain relaxation induced by delamination is also found but with a reduced threshold of Ar plasma reaction time (1 min), while the doping concentration is insensitive to roughness changes. Since the interaction strength between MoS_2_ and Pd is stronger,^41^ the threshold for the delamination would happen earlier than the case of MoS_2_/Au/polyimide. Similarly, strain-induced S–Pd peak shifts of up to 0.1 eV are also observed in the XPS spectra.

Significantly, the S–Au peak shift observed in the S 2p spectrum exhibits a greater magnitude when monolayer MoS_2_ is in contact with Au/HOPG (∼1.2 eV) compared to Au/polyimides (∼0.8 eV), which can be ascribed to the change in doping concentration from 1.2 × 10^13^ to ∼1 × 10^13^ cm^–2^, as shown in Table 1. However, it is important to note that our method of estimating doping concentration based on the strain-corrected shift of the A_1_′ peak may underestimate this difference, as the precise shift in the E′ peak in MoS_2_/Au/HOPG is challenging to determine due to peak asymmetry.^9^ Alternatively, the change in the A_1_′ peak width (3.3 cm^–1^/10^13^ cm^–2^) can be utilized for estimating the doping concentration,^57^ with the peak width of 3.8 cm^–1^ in MoS_2_/HOPG serving as the reference for zero doping. Based on this approach, the doping concentration of MoS_2_/Au/HOPG (width of 5.5 cm^–1^) may actually be approximately three times higher than that of MoS_2_/Au/polyimides (width of 4.3 cm^–1^), suggesting a potential overestimation of strain calculated from the peak shift. Further evidence of the substantially higher doping concentration in MoS_2_/Au/HOPG is observed through the more pronounced asymmetry in the Fano line shape of its E′ peak (Figure 5a).^9^ The decrease in the doping concentration observed in MoS_2_/Au/polyimide can be attributed to the higher surface roughness of polyimides, resulting in a greater average contact distance. This finding aligns with our observations in MoS_2_/Au/polyimide and MoS_2_/Pd/polyimide interfaces with increasing roughness that the doping concentration in Au contact is more sensitive to the contact distance than that in the Pd contact.

The covalent-like quasi-bonding between metal and chalcogen atoms exhibits stronger adhesion than van der Waals interaction but weaker than interlayer bonding.^16^ This weakly covalent bonding nature serves as a basis for modulating the band structure of TMdCs, leading to metallic behavior^14,17^ or partial Fermi level pinning.^6^ As a result, different metal substrates with varying bonding strengths have distinct effects, as shown in our XPS results. The observed dependence of interface interactions on contact materials, surface roughness, and doping concentration holds significant implications. The discrepancies in metal contact resistances^61−65^ and doping conditions^35,66^ reported in TMdCs devices can be attributed to variations in substrate roughness. Particularly when exfoliation or transfer processes are involved during metal contact integration, the substrate roughness becomes crucial. It can alter MoS_2_ morphology, grain size, local strain distribution, and separation between TMdCs and metals, leading to changes in interface interactions and contact resistance. For example, the doping conditions of MoS_2_ have been found to be either n-type^35^ or p-type^66^ when exfoliated by Au surfaces. The roughness condition will also play a critical role in metals with stronger interactions with TMdCs for determining the contact behavior and strain configuration of 2D materials, indicating that the metal type, substrate roughness, and induced strain reinforce the nuanced interplay between material properties and device performance. In the context of MoS_2_-based devices, our findings highlight the necessity of tailoring metal–MoS_2_ interfaces to achieve optimal electronic properties.

## Conclusions

Our study, utilizing HOPG as a substrate, has provided a clearer understanding of the MoS_2_–metal interfacial interaction, which is vital for predicting and controlling device behavior. The variation in XPS peak positions observed for MoS_2_–metal interfaces, especially for MoS_2_/Au/HOPG and MoS_2_/Pd/HOPG, highlights the significant role of metal–sulfur interactions. The degree of electron accumulation at these interfaces is instrumental in dictating their interfacial properties, underscoring the nuanced dynamics of electron transfer and its impact on the overall behavior of these systems.

Furthermore, our experiments shed light on the significant role of surface roughness in determining the morphology and subsequent interfacial interactions of transferred MoS_2_. The presence of strain, as influenced by the underlying surface roughness, has a notable effect on the metal–S interactions at the interface, as revealed through both Raman spectroscopy and XPS.

This work underscores the critical nature of interface engineering in MoS_2_-based devices, emphasizing the significance of optimal substrate choice and treatment. As the field progresses, understanding these intricate relationships will be instrumental in the design and creation of more efficient and reliable devices, facilitating advances in nanoelectronics and optoelectronics.

In conclusion, the intricacies of metal–MoS_2_ interfaces offer promising avenues for future investigations, potentially unlocking transformative improvements in device performance. This study provides a foundation upon which further explorations can be built with the aim of fully harnessing the potential of MoS_2_ in next-generation electronic applications.

## Materials and Methods

### Sample Preparation

The CVD-grown MoS_2_ monolayer was purchased from 2D Semiconductors (CVD-MoS_2_-ML-S) and transferred to the target substrates using a wet etching method.^54^ Poly(methyl methacrylate) (PMMA, 950k, 2.5% in chlorobenzene) was spun onto MoS_2_ on SiO_2_/Si with four edges taped (using Scotch tape) at 3000 rpm for 60 s and then cured at 80 °C for 3 min. The MoS_2_ film was cut into domains of ∼2 × 2 mm^2^ before SiO_2_ was removed by ∼2 M KOH (Merck, SKU 1.05029). Detached PMMA/MoS_2_ films were transferred to DI water multiple times to remove KOH residues. The target substrate was then manually removed from the water below using tweezers to catch the suspended PMMA/MoS_2_ film. To remove the water trapped at the interfaces, we briefly heated and dried. Finally, the sample was dipped into acetone for about 30 min to remove the PMMA and subsequently rinsed in isopropynol. The polyimide film was fabricated by using a spin-coating method (4000 rpm, Dupont, PI2610) developed by the Natural and Medical Sciences Institute (NMI) at the University of Tübingen. The polyimide film thickness was ∼1 μm measured by a profilometer (Veeco Dektak). The Ar plasma was performed on the polyimide film carried by the Si substrate using a Diener plasma tool in the cleanroom. The Pd and Au metal films were deposited on target substrates with a flux of 1 Å s^–1^ and base pressure ≤1 × 10^–6^ mbar using a metal deposition system (Leybold, Univex 450) in the cleanroom. The metal film thickness (∼50 nm) was measured in situ by a crystal sensor and profilometer after deposition.

### X-Ray Photoelectron Spectroscopy

X-Ray photoelectron spectroscopy (XPS) (SPECS) was performed with a nonmonochromatic Mg Kα X-ray source (XR50, *h*ν = 1253.64 eV, 220 W, 10 kV) and a hemispherical electrostatic analyzer (PHOIBOS 150 with MCD-9 spectrometer). The analysis chamber was maintained at base pressure ∼5 × 10^–10^ mbar. The spectra were acquired in sequential mode from the whole sample area. High-resolution spectra of Mo 3d/S 2s (238–224 eV), S 2p (167–159 eV), and Au 4f (92–80 eV) were recorded at a step of 0.05 eV, pass energy of 10 eV, dwell time of 0.1 s, and total scans of 2–6k times. Iris size in the detection path was tuned according to the sample size to be 3–5 mm in diameter. Au 4f_7/2_ at 84 eV was used as our charging reference for all samples. For those samples where the substrate did not contain Au, we deposited gold electrodes next to the MoS_2_. The calibration and linearity of the binding energy scale were confirmed by fixing the positions of Au 4f_7/2_ and Au 4d_5/2_ peaks to 84 and 335.1 eV, respectively, using a Au(111) single crystal sample (MaTecK, Germany). fwhm of Au 4f_7/2_ measured by using a pass energy of 10 eV is ∼0.94 eV. The Pd- and Au-deposited polyimide films and HOPG were connected to the electrical ground by a top metal fixing plate. Before loading into the load-lock chamber, the sample without PMMA was air-exposed for <30 min when assembled on the sample holder. The load-lock chamber was then evacuated overnight to reach a pressure of ∼3 × 10^–8^ mbar before transferring the sample into the analysis chamber.

### XPS Data Analysis

The curve fitting of XPS spectra is done by using CasaXPS.^37^ Peak modeling employs a Shirley-type background and Gaussian–Lorentzian (20%:80%) line shapes. The S 2p (Mo 3d) spin–orbit doublet separation was held constant at 1.2 (3.1) eV and with a fixed 2:1 (3:2) area ratio. The separation between the Mo 3d_5/2_ and S 2s peaks from S–Mo bonds was held constant at 2.8 eV.

### Raman Spectroscopy

Raman spectroscopy (S&I GmbH) was carried out using a 532 nm laser with a spot size of ∼1 μm on the sample, focused by 100× objectives. Incident laser power was kept below <0.12 mW to prevent any thermally induced artifacts. 1200 lines mm^–1^ grating was used. The typical acquisition time was 5 min. Peak parameters were obtained by fitting the spectra using a Lorentzian line shape with CasaXPS.

### Atomic Force Microscopy

Atomic force microscopy (AFM) (Bruker, Dimension Icon) in tapping mode was used to measure the surface morphology and phase shift. All measurements were conducted using the same tip (OLYMPUS, OMCL-AC200TS-R3) to avoid the tip effect in comparing the roughness. Root-mean-square surface roughness was obtained by analyzing the image using Gwyddion.^67^

## Supporting Information Available

Full Raman spectra of MoS_2_/HOPG, MoS_2_/Au/HOPG, and MoS_2_/Pd/HOPG, AFM topography image of Au/polyimides with increasing Ar reaction time, overlay images of AFM topography and phase images of MoS_2_/Au/polyimide, AFM topography and phase images of Au/polyimide and Pd/polyimide with increasing Ar plasma reaction times, AFM topography images of MoS_2_/Au/polyimide treated with Ar for 14 min, AFM topography images of Pd/polyimide before and after transferring the monolayer MoS_2_ with increasing Ar plasma reaction time from 1 and 1.5 to 9 min, AFM topography image of Pd/polyimides with increasing Ar reaction time, overlay images of AFM topography and phase images of MoS_2_/Pd/polyimide, and strain dependence of MoS_2_/Pd interfacial interaction (PDF)
