# Supplementary material for: Investigation of Interface Interactions Between Monolayer MoS2 and Metals: Implications on Strain and Surface Roughness
Source: Langmuir. 2024 Jan 3;40(2):1277–85. doi: 10.1021/acs.langmuir.3c02740 (PMC10795178; doi:10.1021/acs.langmuir.3c02740)
Supplement: Supplementary file 1 — la3c02740_si_001.pdf [file la3c02740_si_001.pdf]

Supporting Information

Investigation of Interface Interactions Between Monolayer MoS_2 and Metals: Implications on Strain and Surface Roughness

Jz-Yuan Juo[1], Klaus Kern[1,2], and Soon Jung Jung[1,*]
[1]Max-Planck-Institut für Festkörperforschung, Heisenbergstraße 1, DE-70569 Stuttgart, Germany
[2]Institut de Physique, École Polytechnique Fédérale de Lausanne, CH-1015 Lausanne, Switzerland
*corresponding author

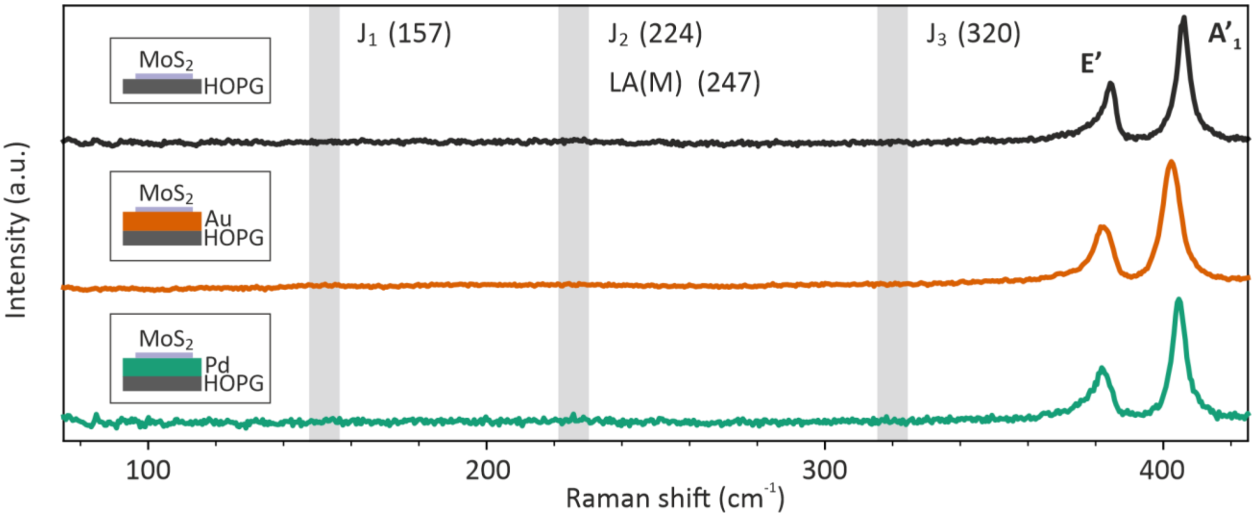

FIG. S1. Absence of modes for 1T phase and defects in the normalized representative Raman spectra of MoS_2/HOPG, MoS_2/Au/HOPG, and MoS_2/Pd/HOPG.

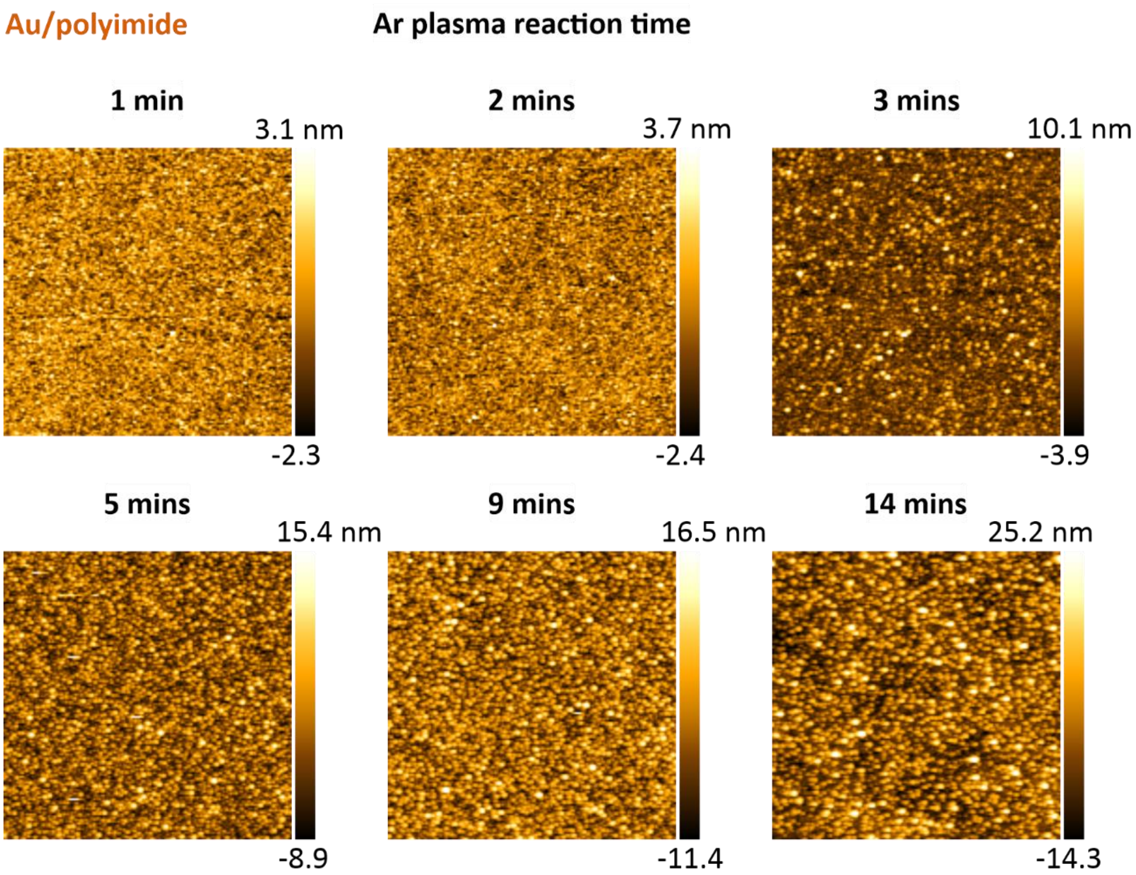

FIG. S2. Representative AFM topography image of Au/polyimides with increasing Ar reaction time. The image size is 5 x 5 μm^2.

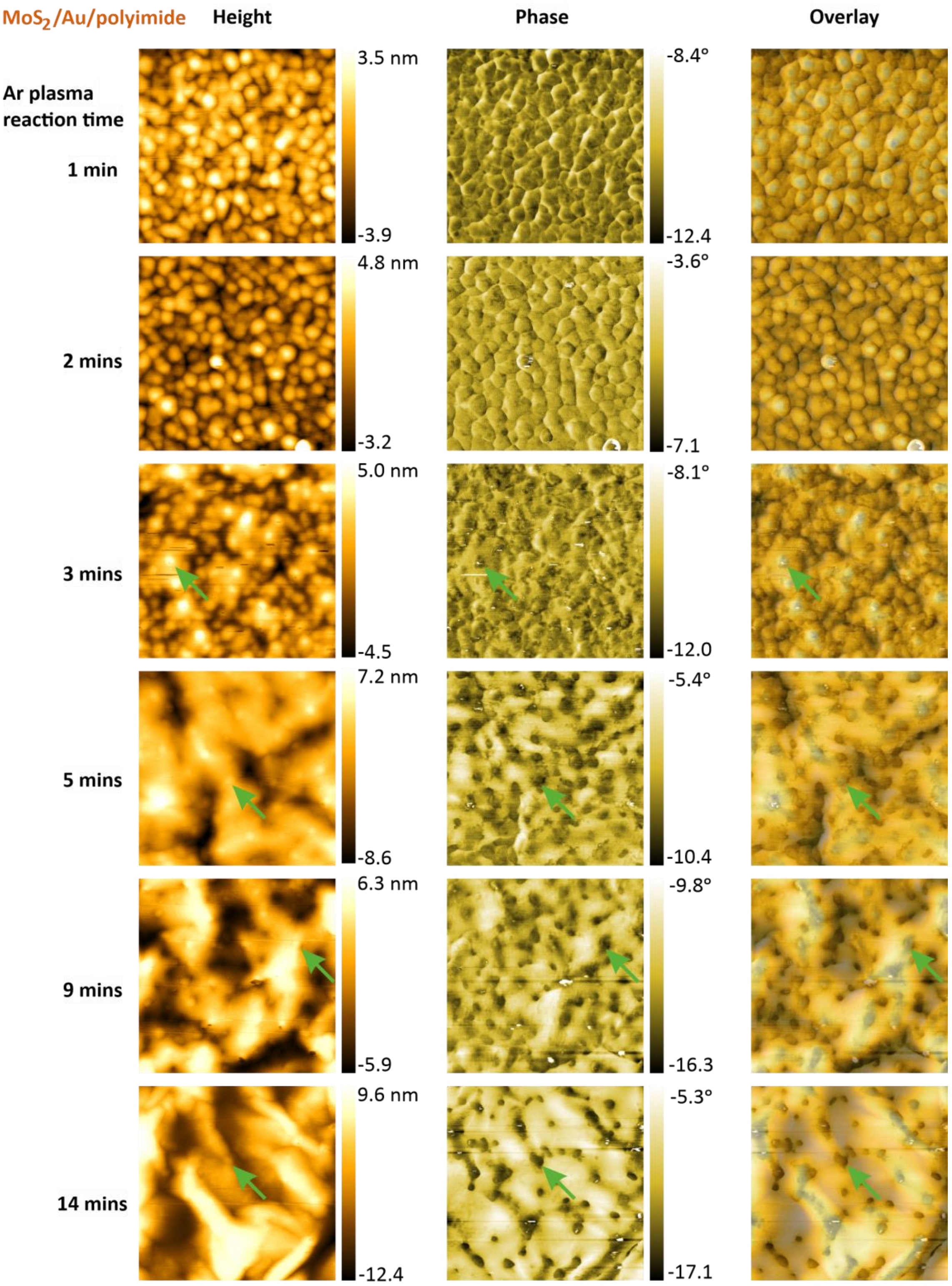

FIG. S3. Overlay images of AFM topography and phase images of MoS_2/Au/polyimide. The black dot area seen in the phase images match with the supported area in height images. The image size is 0.5 x 0.5 μm^2.

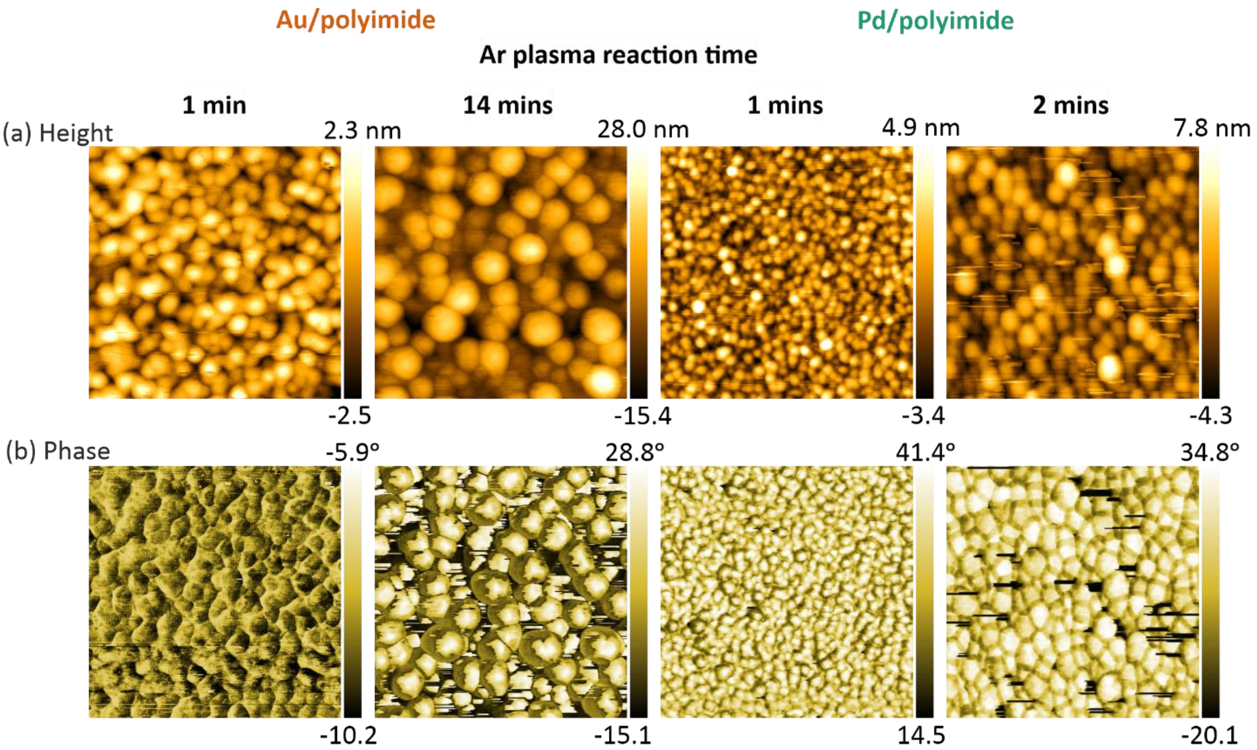

FIG. S4. Representative AFM (a) topography and (b) phase images of Au/polyimide and Pd/polyimide with increasing Ar plasma reaction times. The image size is 0.5 x 0.5 μm^2.

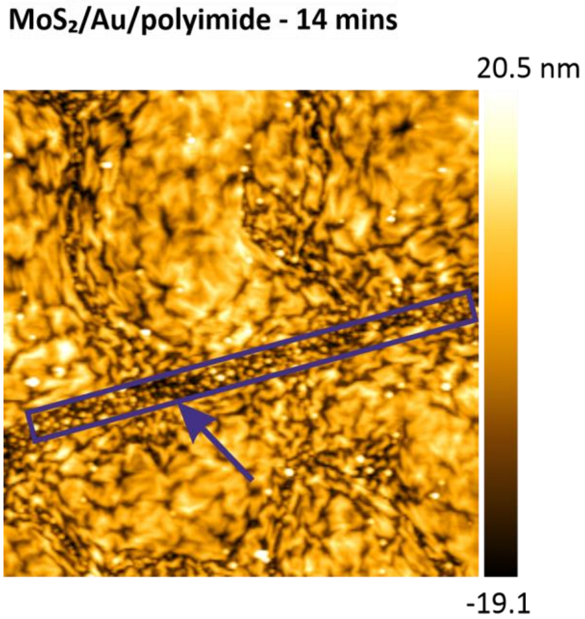

FIG. S5. Representative AFM topography images of MoS_2/Au/polyimide treated with Ar for 14 mins. The image size is 5 x 5 μm^2. The visible cracks are marked.

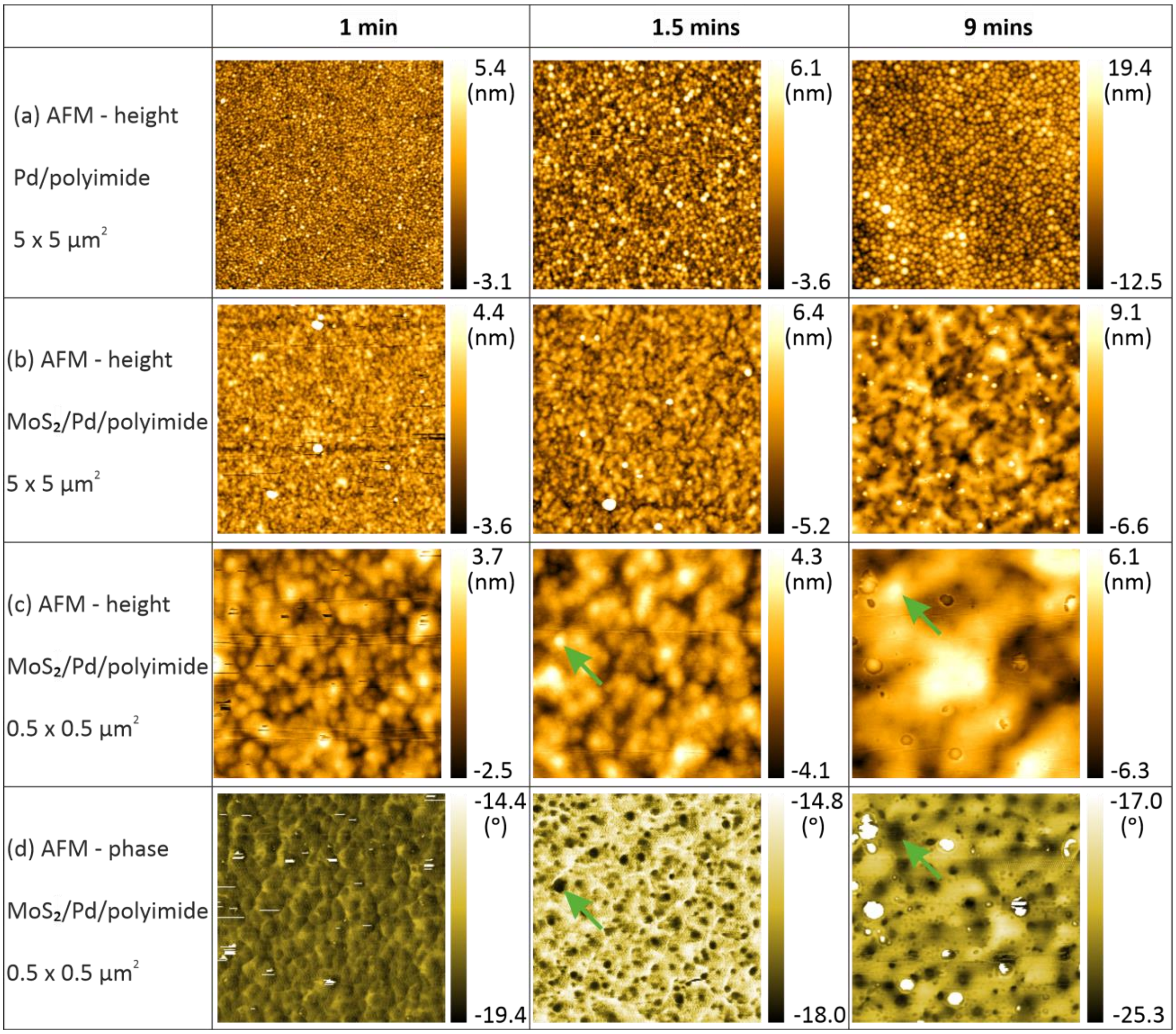

FIG. S6. Representative AFM topography images of Pd/polyimide (a) before and (b) after transferring the monolayer MoS_2 with increasing Ar plasma reaction time from 1 min, 1.5 mins, to 9 mins. Higher resolution AFM (c) topography and (d) simultaneously measured phase images of MoS_2/Pd/polyimide.

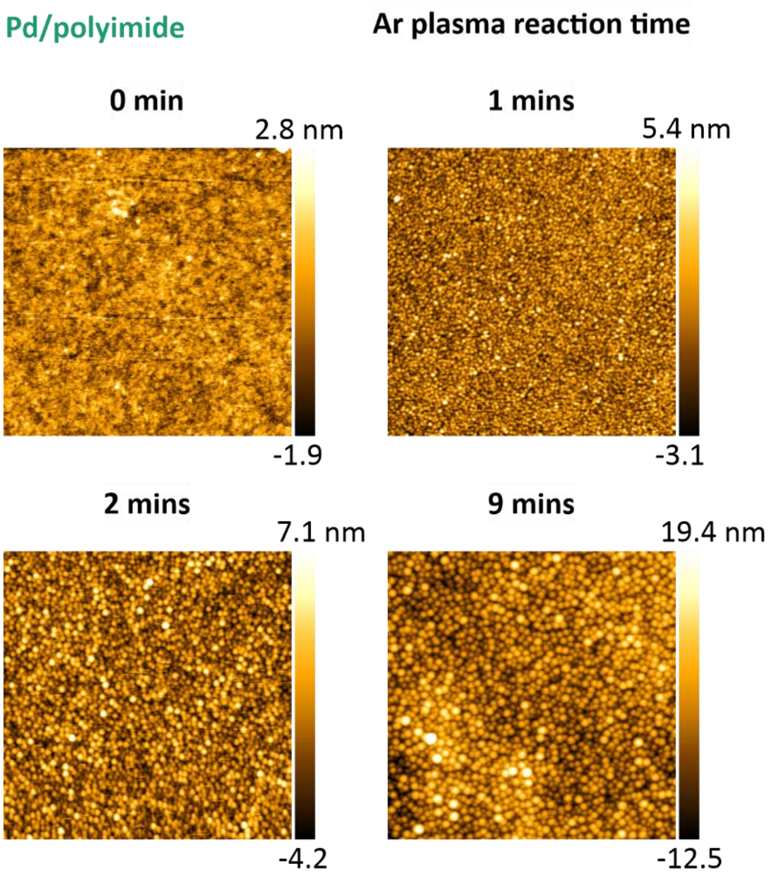

FIG. S7. Representative AFM topography image of Pd/polyimides with increasing Ar reaction time. The image size is 5 x 5 μm^2.

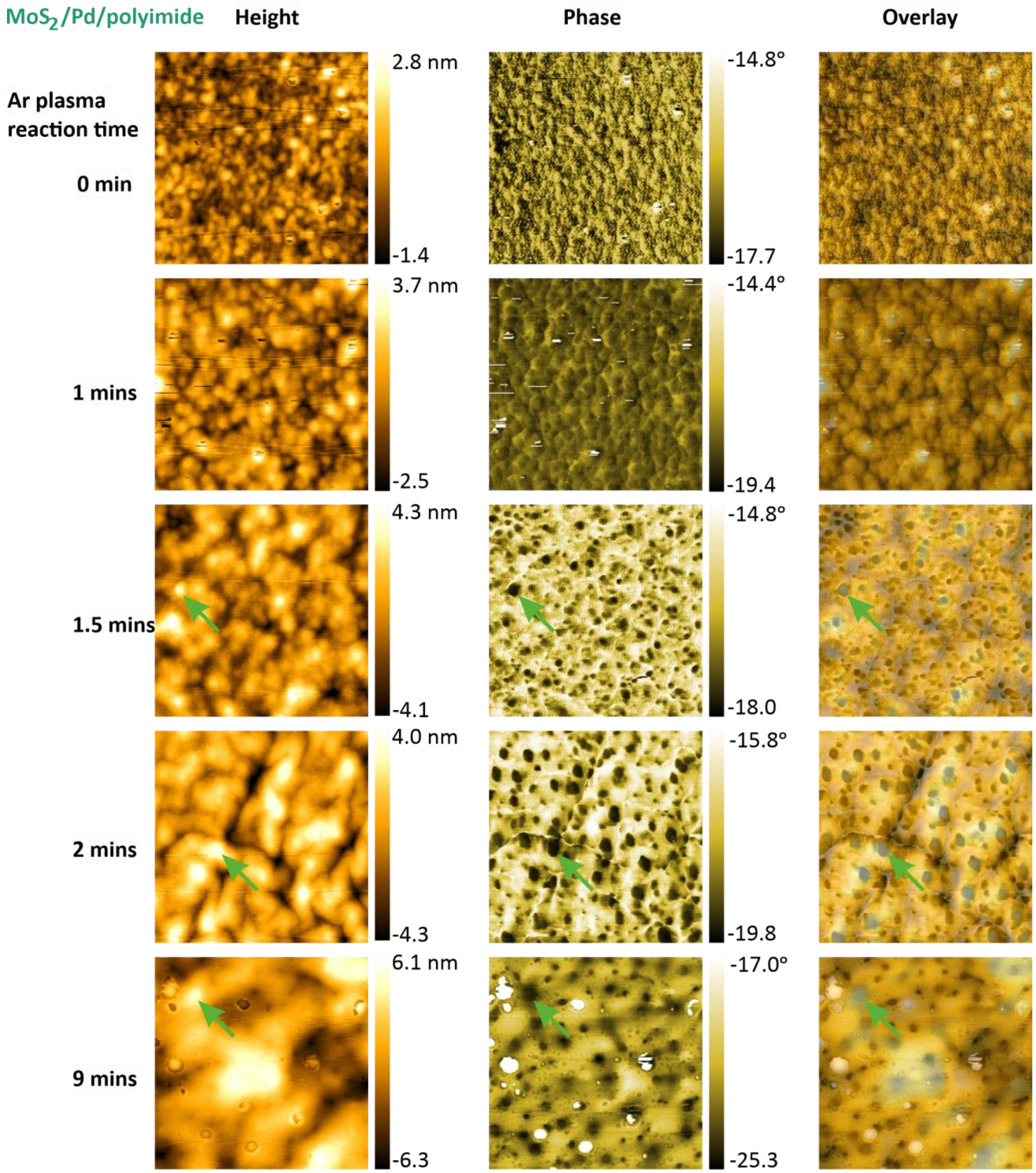

FIG. S8. Overlay images of AFM topography and phase images of MoS_2/Pd/polyimide. The black dot area seen in the phase images match with the supported area in height images. The image size is 0.5 x 0.5 μm^2.

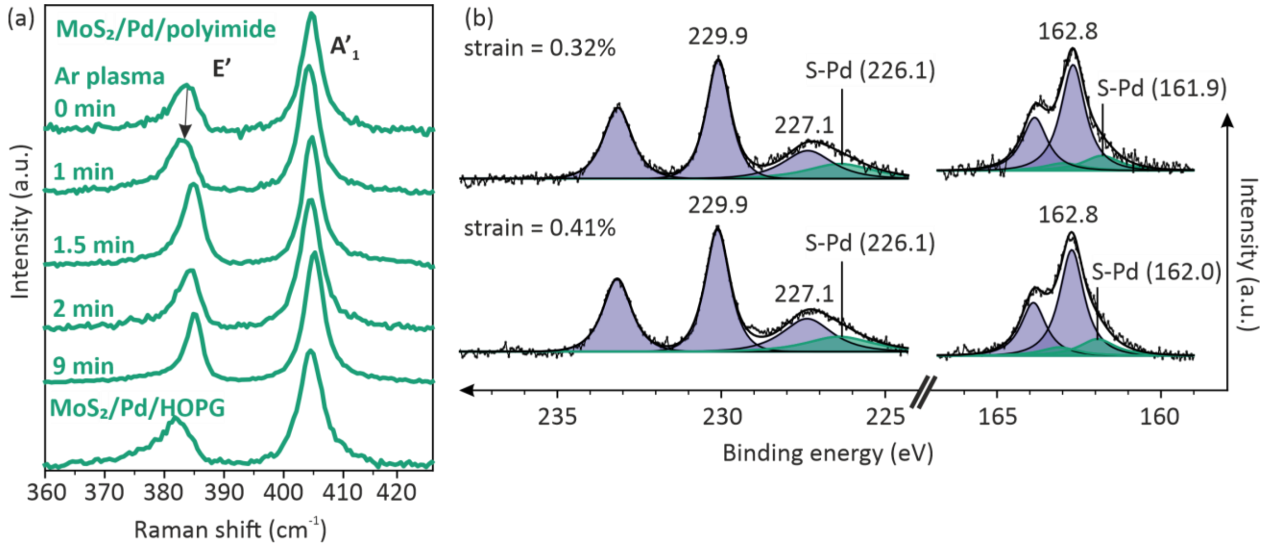

FIG. S9. Strain dependence of MoS_2/Pd interfacial interaction. (a) Normalized representative Raman spectra of monolayer MoS_2/Pd/polyimides treated with increasing Ar plasma reaction time. (b) Normalized XPS spectra of the Mo 3d, S 2s, and S 2p core levels of monolayer MoS_2/Pd/polyimide with increasing strain, which are extracted from their Raman spectra, as shown in Table 1. Peaks from intrinsic MoS_2 are marked as purple. S 2s and S 2p peaks for S-Pd are marked as green.
